# Supplementary material for: Integron activity accelerates the evolution of antibiotic resistance
Source: eLife. 2021 Feb 26;10:e62474. doi: 10.7554/eLife.62474 (PMC8024014; doi:10.7554/eLife.62474)
Supplement: Supplementary file 4. [file elife-62474-supp4.doc]

**Table S5 – List of duplicated cassettes from the INTEGRALL database.** Cassettes found in duplicates within the list of numbered class 1 integrons from the INTEGRALL database (from the July 30th November 2017 update). ‘Arrays count’ represents the number of different arrays in which a duplicated cassette was observed.

| Duplicated cassettes | Arrays count |
| --- | --- |
| aacA29b | 1 |
| aacA29e | 1 |
| aacA4 | 7 |
| aacA4'-17 | 1 |
| aacA4'-3 | 5 |
| aacA4-5 | 2 |
| aacA48 | 1 |
| aacA7 | 3 |
| aadA1a | 1 |
| aadA1b | 5 |
| aadA2 | 1 |
| aadA7 | 1 |
| aadB | 5 |
| blaIMP-1 | 1 |
| blaOXA-2 | 3 |
| blaVIM-1 | 1 |
| blaVIM-4 | 1 |
| fosL | 1 |
| gcu110 | 1 |
| gcu114 | 1 |
| gcu2b | 1 |
| gcuD | 1 |
| gcuP | 4 |
| qacG | 1 |
